# Retraction Note: Sign language recognition using the fusion of image and hand landmarks through multi-headed convolutional neural network

**DOI:** 10.1038/s41598-025-85429-w

**Published:** 2025-01-08

**Authors:** Refat Khan Pathan, Munmun Biswas, Suraiya Yasmin, Mayeen Uddin Khandaker, Mohammad Salman, Ahmed A. F. Youssef

**Affiliations:** 1 Department of Computing and Information Systems, School of Engineering and Technology, Sunway University, 47500 Bandar Sunway, Selangor Malaysia; 2 Department of Computer Science and Engineering, BGC Trust University Bangladesh, Chittagong, 4381 Bangladesh; 3 Department of Computer and Information Science, Graduate School of Engineering, Tokyo University of Agriculture and Technology, Koganei, Tokyo 184-0012 Japan; 4 Centre for Applied Physics and Radiation Technologies, School of Engineering and Technology, Sunway University, 47500 Bandar Sunway, Selangor Malaysia; 5 Faculty of Graduate Studies, Daffodil International University, Daffodil Smart City, Birulia, Savar, Dhaka, 1216 Bangladesh; 6 College of Engineering and Technology, American University of the Middle East, Egaila, Kuwait

Retraction of: *Scientific Reports* 10.1038/s41598-023-43852-x, published online 09 October 2023

The Editors have retracted this Article because it contains a number of nonstandard phrases. These phrases may have been introduced as the result of editing software to reduce overlap with previously published articles. The Editors therefore no longer have confidence in the reliability and the original contribution of this Article.

Authors Refat Khan Pathan and Mohammad Salman disagree with this retraction. The other Authors have not responded to correspondence regarding this retraction.

